# Supplementary material for: Transcriptomic Insights into Mechanisms of Early Seed Maturation in the Garden Pea (Pisum sativum L.)
Source: Cells. 2020 Mar 23;9(3):779. doi: 10.3390/cells9030779 (PMC7140803; doi:10.3390/cells9030779)
Supplement: Supplementary file 1 [file cells-09-00779-s001.zip › Supplementary materials/FileS4.zip!/FileS3/TFs - Results/Summary Sheets - Modules/K-Means Cluster/Report.pdf]

K−Means Cluster

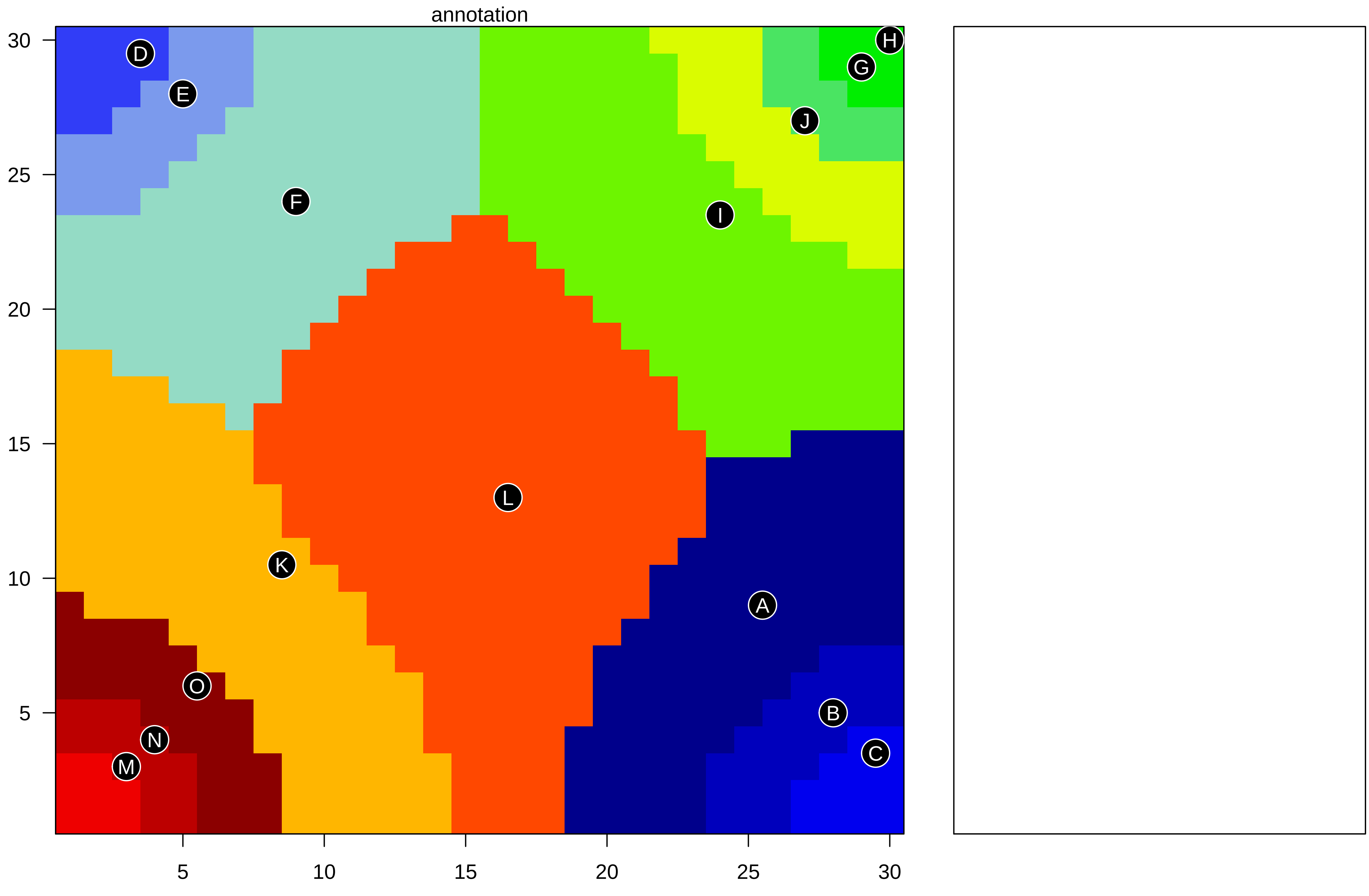

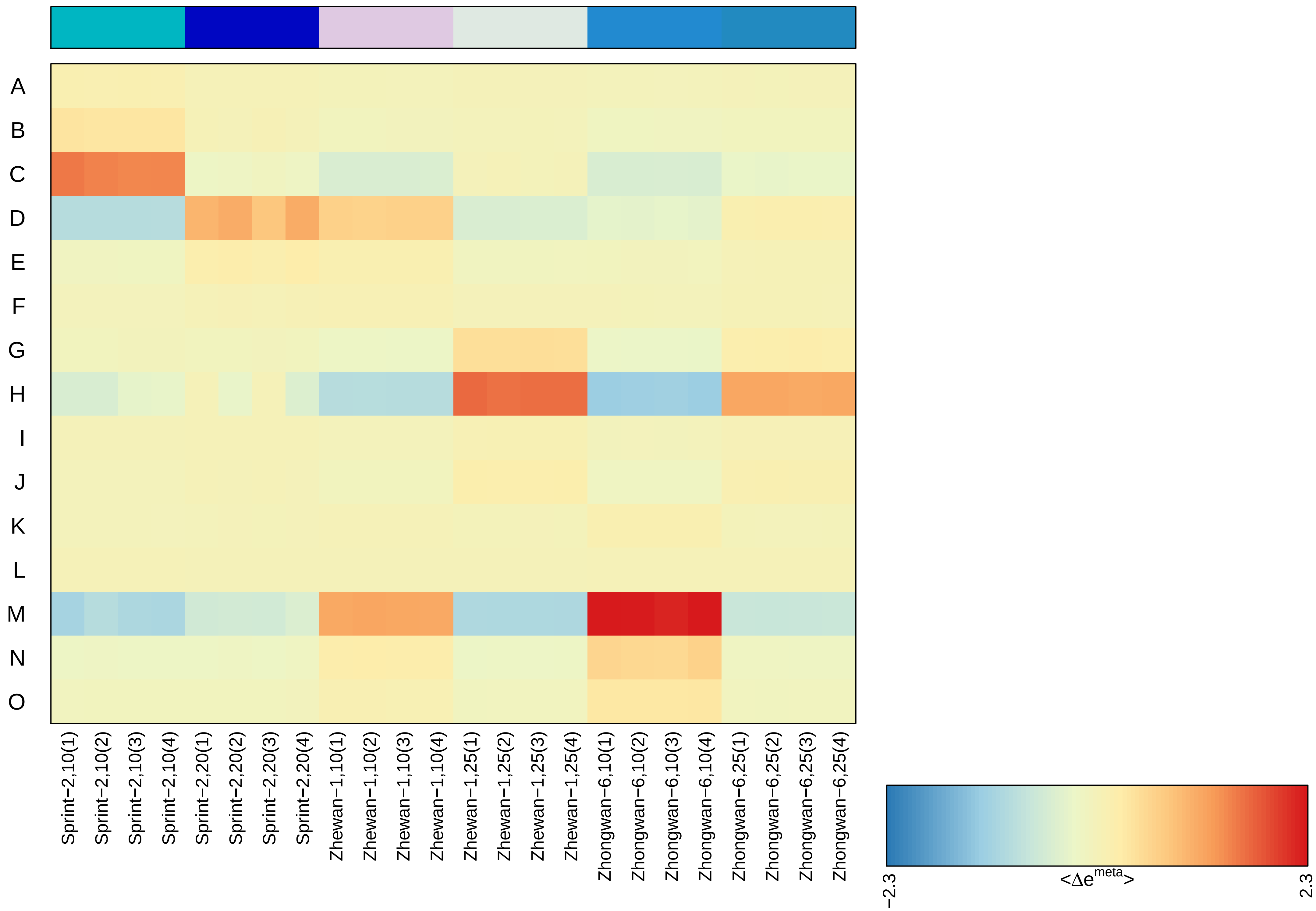
A
B
C
D
E
F
G
H
I
J
K
L
M
N
O
Sprint−2,10(1)
Sprint−2,10(2)
Sprint−2,10(3)
Sprint−2,10(4)
Sprint−2,20(1)
Sprint−2,20(2)
Sprint−2,20(3)
Sprint−2,20(4)
Zhewan−1,10(1)
Zhewan−1,10(2)
Zhewan−1,10(3)
Zhewan−1,10(4)
Zhewan−1,25(1)
Zhewan−1,25(2)
Zhewan−1,25(3)
Zhewan−1,25(4)
Zhongwan−6,10(1)
Zhongwan−6,10(2)
Zhongwan−6,10(3)
Zhongwan−6,10(4)
Zhongwan−6,25(1)
Zhongwan−6,25(2)
Zhongwan−6,25(3)
Zhongwan−6,25(4)
−2.3
$\langle \Delta e^{meta} \rangle$
2.3

K−Means Cluster

Spot Summary: A

metagenes = 103
genes = 179

<r> metagenes = 0.87
<r> genes = 0.53
beta: r2= 0.08 / log p= −0.74

samples with spot = 0 (0 %)

Overview Map

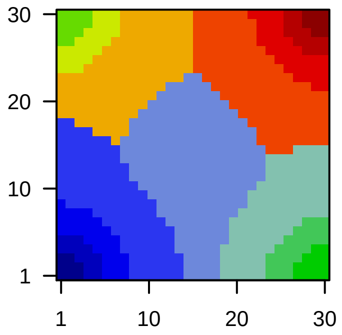

Spot

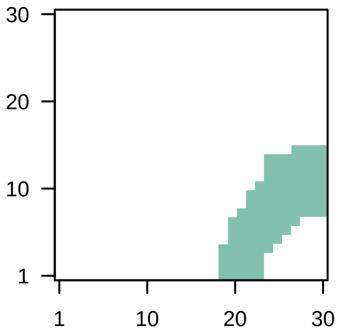

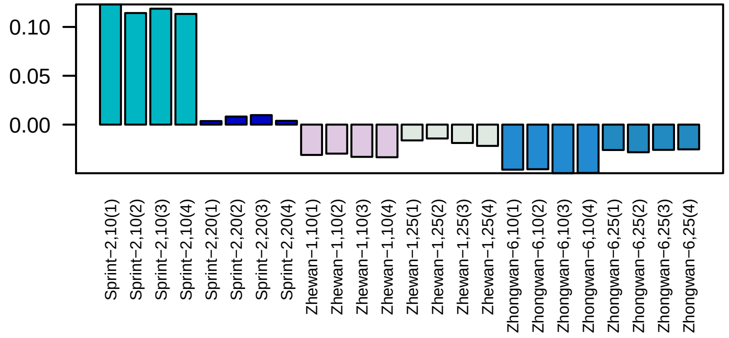

Spot Genelist

Rank	ID	max e	min e	r	Symbol	Description
1	TRINITY_DN8	0.38	−0.17	0.63		
2	TRINITY_DN3	0.35	−0.26	0.29		
3	TRINITY_DN9	0.34	−0.21	0.35		
4	TRINITY_DN5	0.29	−0.38	0.58		
5	TRINITY_DN2	0.29	−0.13	0.87		
6	TRINITY_DN2	0.28	−0.17	0.76		
7	TRINITY_DN3	0.28	−0.41	0.59		
8	TRINITY_DN1	0.28	−0.07	0.86		
9	TRINITY_DN2	0.28	−0.16	0.49		
10	TRINITY_DN5	0.27	−0.19	0.38		
11	TRINITY_DN7	0.27	−0.16	0.63		
12	TRINITY_DN3	0.26	−0.2	0.5		
13	TRINITY_DN1	0.25	−0.1	0.83		
14	TRINITY_DN5	0.25	−0.14	0.9		
15	TRINITY_DN8	0.25	−0.15	0.78		
16	TRINITY_DN1	0.24	−0.2	0.78		
17	TRINITY_DN3	0.24	−0.06	0.86		
18	TRINITY_DN5	0.24	−0.07	0.89		
19	TRINITY_DN1	0.23	−0.11	0.52		
20	TRINITY_DN1	0.23	−0.1	0.65		

K−Means Cluster

Spot Summary: B

metagenes = 26
genes = 122

<r> metagenes = 0.98
<r> genes = 0.61
beta: r2= 0.59 / log p= −4.92

samples with spot = 0 (0 %)

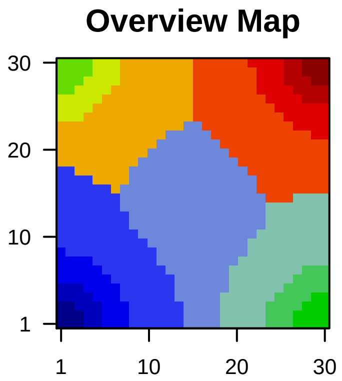

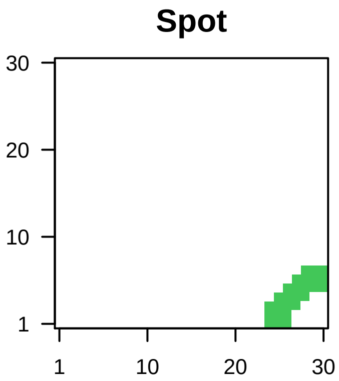

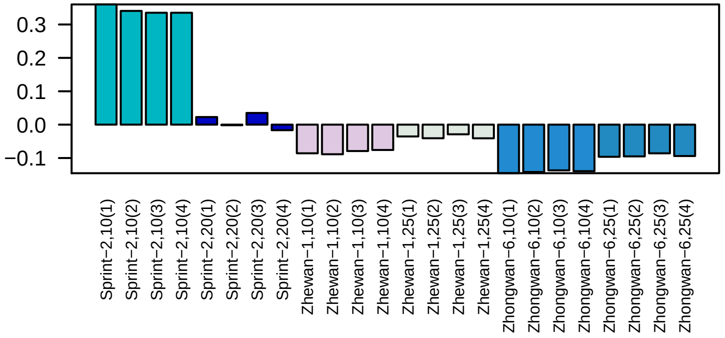

Spot Genelist

Rank	ID	max e	min e	r	Symbol	Description
1	TRINITY_DN5	2.18	−1.08	0.29		
2	TRINITY_DN4	1.76	−1.39	0.33		
3	TRINITY_DN9	1.42	−0.37	0.47		
4	TRINITY_DN3	1.4	−0.34	0.48		
5	TRINITY_DN1	1.29	−0.81	0.39		
6	TRINITY_DN1	1.18	−0.43	0.49		
7	TRINITY_DN3	1.11	−1	0.54		
8	TRINITY_DN1	0.79	−0.51	0.55		
9	TRINITY_DN1	0.72	−0.27	0.65		
10	TRINITY_DN2	0.7	−0.15	0.83		
11	TRINITY_DN6	0.7	−1.26	0.19		
12	TRINITY_DN3	0.67	−0.57	0.48		
13	TRINITY_DN1	0.64	−0.1	0.92		
14	TRINITY_DN4	0.62	−0.8	0.57		
15	TRINITY_DN4	0.62	−0.24	0.71		
16	TRINITY_DN1	0.61	−0.11	0.95		
17	TRINITY_DN2	0.56	−0.21	0.94		
18	TRINITY_DN9	0.54	−0.11	0.93		
19	TRINITY_DN3	0.54	−0.4	0.84		
20	TRINITY_DN4	0.54	−0.31	0.96		

K−Means Cluster

Spot Summary: C

metagenes = 13
genes = 92

<r> metagenes = 1
<r> genes = 0.73
beta: r2= 3.14 / log p= −Inf

samples with spot = 4 (16.7 %)
Sprint−2(10) : 4 (100 %)

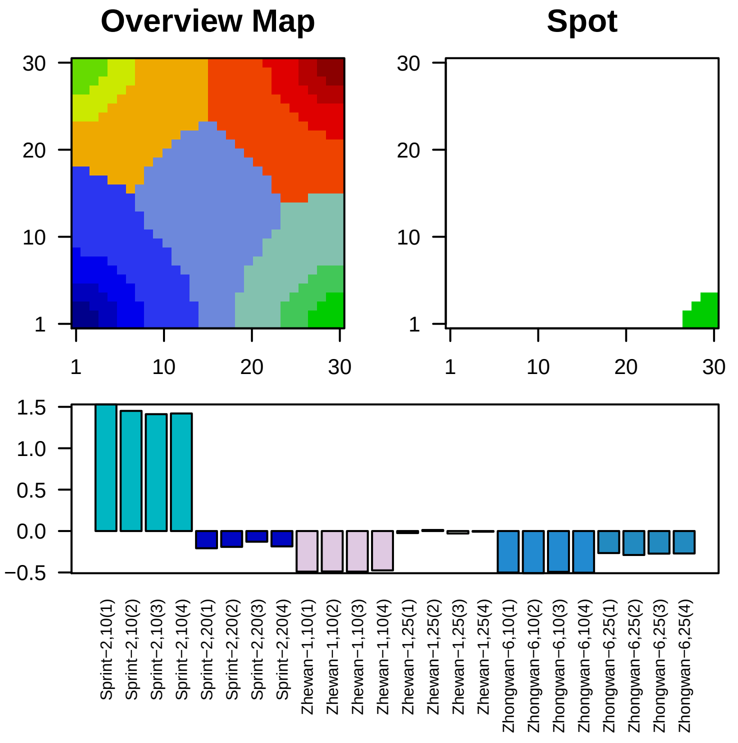

Spot Genelist

Rank	ID	max e	min e	r	Description	Symbol
1	TRINITY_DN5	8.61	−1.68	0.96		
2	TRINITY_DN2	7.6	−8.44	0.83		
3	TRINITY_DN1	7.51	−1.97	0.98		
4	TRINITY_DN1	4.85	−1.19	0.96		
5	TRINITY_DN1	4.67	−2.15	0.92		
6	TRINITY_DN6	4.43	−1.66	0.85		
7	TRINITY_DN9	3.91	−0.84	0.96		
8	TRINITY_DN7	3.84	−2.46	0.47		
9	TRINITY_DN1	3.43	−1.66	0.82		
10	TRINITY_DN1	3.36	−2.28	0.87		
11	TRINITY_DN6	3.29	−1.71	0.92		
12	TRINITY_DN1	3.27	−0.38	0.79		
13	TRINITY_DN7	3	−0.92	0.92		
14	TRINITY_DN2	2.98	−3.37	0.68		
15	TRINITY_DN1	2.98	−1.06	0.98		
16	TRINITY_DN7	2.89	−0.48	0.96		
17	TRINITY_DN3	2.65	−0.56	0.96		
18	TRINITY_DN1	2.6	−0.83	0.98		
19	TRINITY_DN5	2.52	−1.4	0.83		
20	TRINITY_DN5	2.4	−1.02	0.95		

K−Means Cluster

Spot Summary: D

metagenes = 13
genes = 67

<r> metagenes = 1
<r> genes = 0.61
beta: r2= 2.05 / log p= −Inf

samples with spot = 8 (33.3 %)
Sprint−2(20) : 4 (100 %)
Zhewan−1(10) : 4 (100 %)

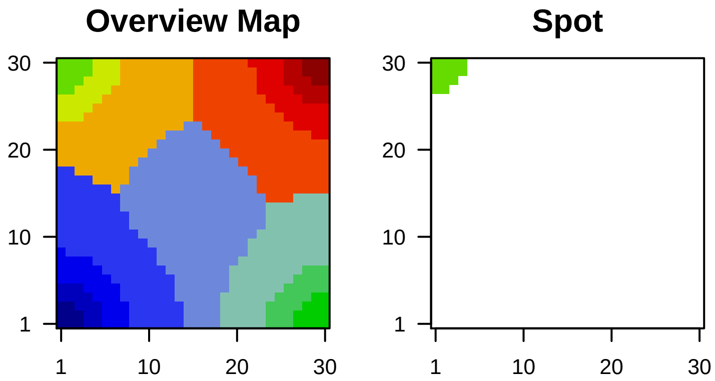

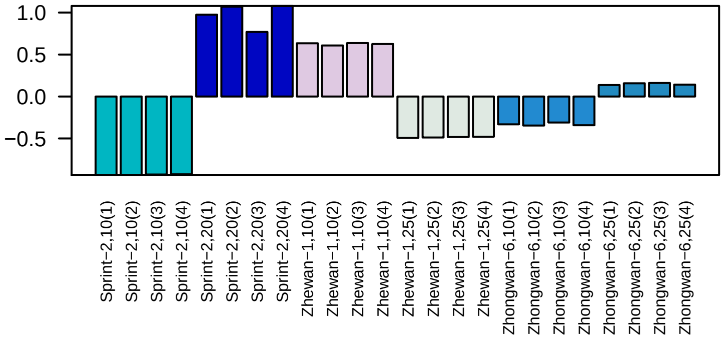

Spot Genelist

Rank	ID	max e	min e	r	Symbol	Description
1	TRINITY_DN4	8.82	−8.66	0.92		
2	TRINITY_DN4	8.39	−5.76	0.92		
3	TRINITY_DN4	5.37	−4.32	0.97		
4	TRINITY_DN1	3.77	−2.56	0.83		
5	TRINITY_DN3	3.65	−1.82	0.75		
6	TRINITY_DN5	3.42	−2.06	0.88		
7	TRINITY_DN4	2.8	−1.11	0.77		
8	TRINITY_DN2	2.61	−1.75	0.82		
9	TRINITY_DN5	2.36	−2.06	0.82		
10	TRINITY_DN5	2.24	−1.8	0.86		
11	TRINITY_DN4	2.18	−1.82	0.81		
12	TRINITY_DN2	2.08	−1.08	0.9		
13	TRINITY_DN1	2.07	−0.94	0.73		
14	TRINITY_DN4	1.85	−1.11	0.73		
15	TRINITY_DN5	1.76	−0.46	0.7		
16	TRINITY_DN1	1.73	−1.06	0.87		
17	TRINITY_DN1	1.71	−1.55	0.67		
18	TRINITY_DN5	1.6	−1.18	0.58		
19	TRINITY_DN9	1.54	−0.51	0.43		
20	TRINITY_DN1	1.54	−0.9	0.98		

K−Means Cluster

Spot Summary: E

metagenes = 26
genes = 78

<r> metagenes = 0.97
<r> genes = 0.48
beta: r2= 0.37 / log p= −2.76

samples with spot = 0 (0 %)

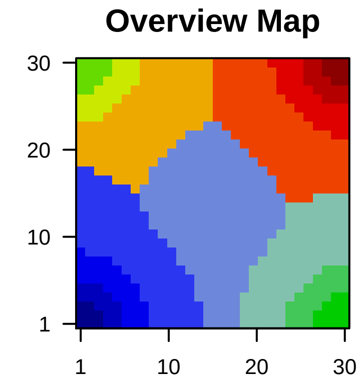

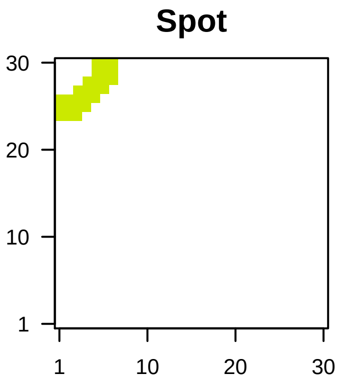

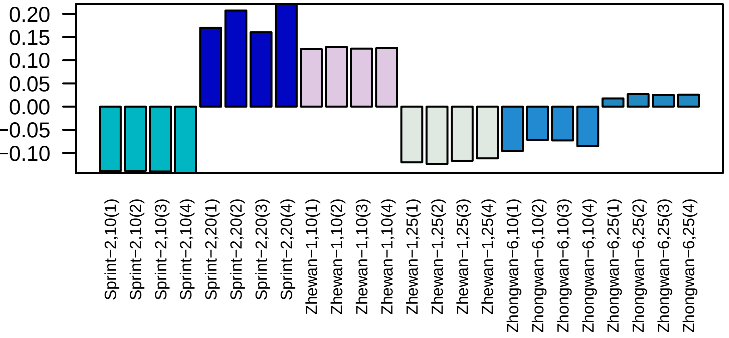

Spot Genelist

Rank	ID	max e	min e	r	Symbol	Description
1	TRINITY_DN7	0.99	−0.38	0.56		
2	TRINITY_DN1	0.85	−0.22	0.52		
3	TRINITY_DN4	0.79	−0.34	0.63		
4	TRINITY_DN7	0.77	−0.41	0.52		
5	TRINITY_DN6	0.66	−0.23	0.54		
6	TRINITY_DN4	0.61	−0.51	0.61		
7	TRINITY_DN2	0.58	−0.25	0.47		
8	TRINITY_DN1	0.57	−0.22	0.61		
9	TRINITY_DN4	0.55	−0.38	0.64		
10	TRINITY_DN1	0.54	−0.27	0.51		
11	TRINITY_DN6	0.53	−0.21	0.74		
12	TRINITY_DN9	0.51	−0.4	0.6		
13	TRINITY_DN4	0.49	−0.22	0.78		
14	TRINITY_DN7	0.46	−0.3	0.64		
15	TRINITY_DN7	0.46	−0.38	0.67		
16	TRINITY_DN1	0.46	−0.14	0.81		
17	TRINITY_DN1	0.44	−0.23	0.83		
18	TRINITY_DN2	0.44	−0.35	0.6		
19	comp34162_c	0.44	−0.27	0.62		
20	TRINITY_DN5	0.44	−0.35	0.59		

K−Means Cluster

Spot Summary: F

metagenes = 133
genes = 189

<r> metagenes = 0.77
<r> genes = 0.31
beta: r2= 0.04 / log p= −0.45

samples with spot = 0 (0 %)

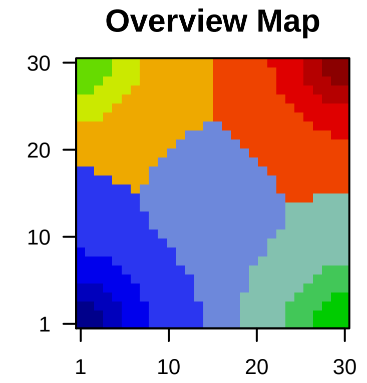

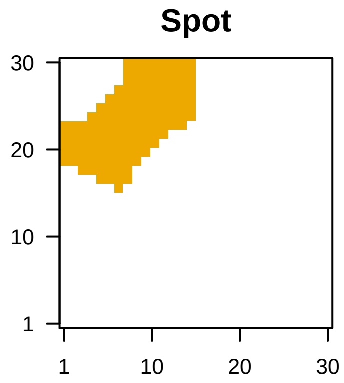

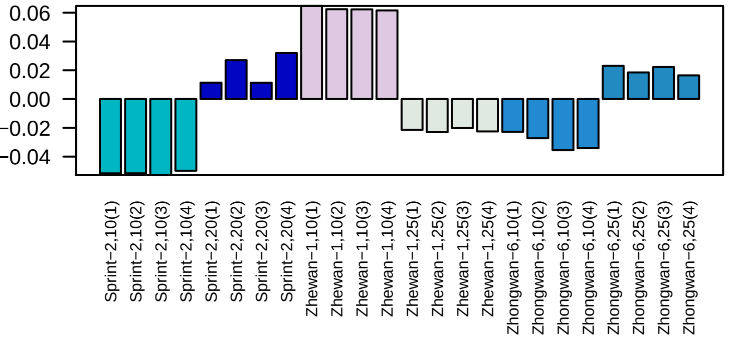

Spot Genelist

Rank	ID	max e	min e	r	Symbol	Description
1	TRINITY_DN5	0.49	−0.35	0.38		
2	TRINITY_DN5	0.41	−0.46	0.48		
3	TRINITY_DN7	0.39	−0.2	0.6		
4	TRINITY_DN1	0.36	−0.3	0.22		
5	TRINITY_DN3	0.31	−0.27	0.6		
6	TRINITY_DN6	0.31	−0.25	0.12		
7	TRINITY_DN4	0.3	−0.22	0.4		
8	TRINITY_DN8	0.3	−0.24	0.37		
9	TRINITY_DN2	0.29	−0.13	0.54		
10	TRINITY_DN7	0.29	−0.31	0.5		
11	TRINITY_DN7	0.29	−0.13	0.18		
12	comp67792_c	0.29	−0.03	0.58		
13	TRINITY_DN1	0.28	−0.2	0.56		
14	TRINITY_DN5	0.28	−0.21	0.51		
15	TRINITY_DN2	0.28	−0.21	0.24		
16	comp24437_c	0.27	−0.1	0.71		
17	TRINITY_DN8	0.26	−0.13	0.77		
18	TRINITY_DN1	0.26	−0.38	0.54		
19	TRINITY_DN4	0.26	−0.06	0.64		
20	TRINITY_DN1	0.26	−0.17	0.31		

K−Means Cluster

Spot Summary: G

metagenes = 14
genes = 41

<r> metagenes = 0.99
<r> genes = 0.65
beta: r2= 1.36 / log p= −Inf

samples with spot = 0 (0 %)

Overview Map

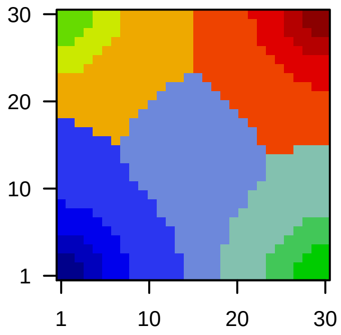

Spot

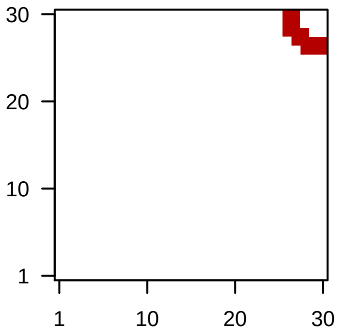

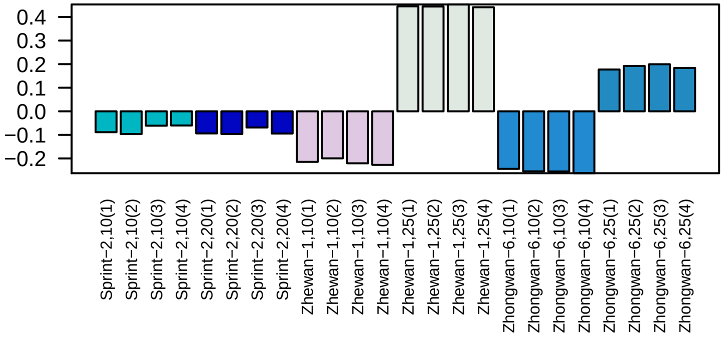

Spot Genelist

Rank	ID	max e	min e	r	Symbol	Description
1	TRINITY_DN8	1.22	−0.87	0.56		
2	TRINITY_DN1	0.98	−0.49	0.7		
3	TRINITY_DN8	0.89	−0.36	0.81		
4	TRINITY_DN7	0.84	−0.57	0.83		
5	TRINITY_DN3	0.81	−0.21	0.57		
6	TRINITY_DN8	0.77	−0.44	0.87		
7	TRINITY_DN1	0.76	−0.6	0.78		
8	TRINITY_DN3	0.72	−0.38	0.62		
9	TRINITY_DN1	0.71	−0.17	0.84		
10	TRINITY_DN4	0.71	−0.18	0.87		
11	TRINITY_DN5	0.7	−0.39	0.97		
12	TRINITY_DN1	0.69	−0.46	0.76		
13	TRINITY_DN5	0.66	−0.52	0.67		
14	TRINITY_DN3	0.66	−0.47	0.75		
15	TRINITY_DN2	0.65	−0.27	0.91		
16	TRINITY_DN8	0.63	−0.38	0.74		
17	TRINITY_DN9	0.63	−0.38	0.91		
18	TRINITY_DN1	0.63	−0.29	0.98		
19	TRINITY_DN1	0.61	−0.35	0.57		
20	TRINITY_DN9	0.61	−0.29	0.82		

K−Means Cluster

Spot Summary: H

metagenes = 8
genes = 54

<r> metagenes = 1
<r> genes = 0.67
beta: r2= 4.63 / log p= −Inf

samples with spot = 8 (33.3 %)
Zhewan−1(25) : 4 (100 %)
Zhongwan−6(25) : 4 (100 %)

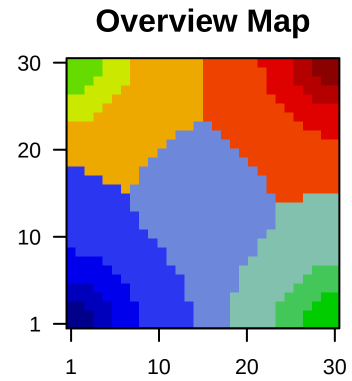

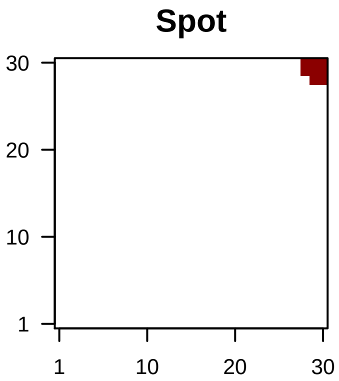

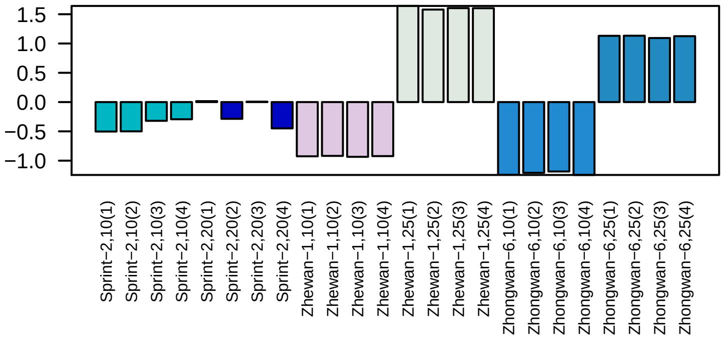

Spot Genelist

Rank	ID	max e	min e	r	Description	Symbol
1	TRINITY_DN1	10.4	−3.02	0.81		
2	TRINITY_DN4	7.91	−7.93	0.93		
3	TRINITY_DN4	7.74	−4.91	0.8		
4	TRINITY_DN1	7.72	−3.91	0.79		
5	TRINITY_DN2	5.9	−1.92	0.77		
6	TRINITY_DN2	4.81	−3.98	0.96		
7	TRINITY_DN5	4.22	−2.39	0.98		
8	TRINITY_DN2	4.11	−3.63	0.9		
9	TRINITY_DN3	3.31	−3.6	0.55		
10	TRINITY_DN1	3.23	−1.54	0.92		
11	TRINITY_DN8	2.99	−2.22	0.83		
12	TRINITY_DN7	2.62	−3.66	0.56		
13	TRINITY_DN6	2.53	−0.84	0.86		
14	TRINITY_DN1	2.27	−1	0.94		
15	TRINITY_DN2	2.25	−1.48	0.58		
16	TRINITY_DN4	2.14	−0.47	0.6		
17	TRINITY_DN1	2.09	−1.85	0.76		
18	TRINITY_DN9	2.04	−0.73	0.84		
19	TRINITY_DN9	1.98	−1.08	0.87		
20	TRINITY_DN4	1.92	−4.32	0.64		

K−Means Cluster

Spot Summary: I

metagenes = 136
genes = 158

<r> metagenes = 0.83
<r> genes = 0.37
beta: r2= 0.05 / log p= −0.5

samples with spot = 0 (0 %)

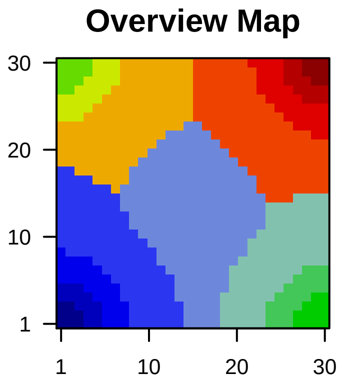

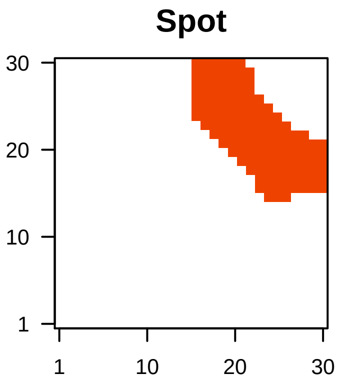

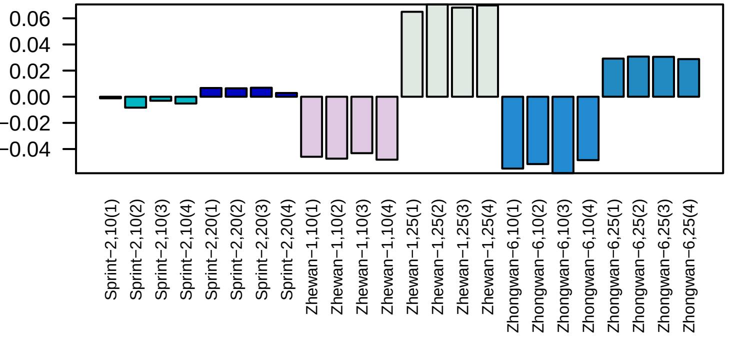

Spot Genelist

Rank	ID	max e	min e	r	Symbol	Description
1	TRINITY_DN1	0.54	−0.29	0.55		
2	TRINITY_DN2	0.39	−0.09	0.28		
3	TRINITY_DN9	0.37	−0.28	0.31		
4	TRINITY_DN1	0.32	−0.37	0.56		
5	TRINITY_DN3	0.32	−0.2	0.36		
6	TRINITY_DN2	0.29	−0.11	0.43		
7	TRINITY_DN2	0.28	−0.05	0.21		
8	TRINITY_DN3	0.27	−0.07	0.37		
9	TRINITY_DN7	0.27	−0.18	0.39		
10	TRINITY_DN7	0.26	−0.07	0.57		
11	TRINITY_DN7	0.25	−0.21	0.5		
12	TRINITY_DN4	0.24	−0.14	0.45		
13	TRINITY_DN2	0.24	−0.5	0.52		
14	TRINITY_DN3	0.23	−0.29	0.62		
15	TRINITY_DN4	0.23	−0.27	0.51		
16	TRINITY_DN1	0.23	−0.12	0.52		
17	TRINITY_DN3	0.22	−0.23	0.33		
18	TRINITY_DN5	0.22	−0.19	0.44		
19	TRINITY_DN3	0.22	−0.17	0.39		
20	TRINITY_DN1	0.22	−0.3	0.64		

K−Means Cluster

Spot Summary: J

metagenes = 35
genes = 128

<r> metagenes = 0.95
<r> genes = 0.49
beta: r2= 0.35 / log p= −2.62

samples with spot = 0 (0 %)

Overview Map

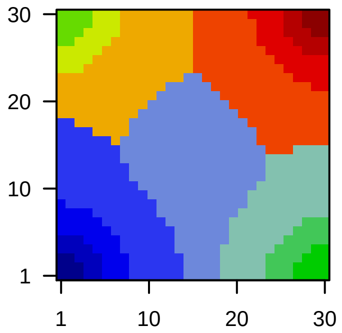

Spot

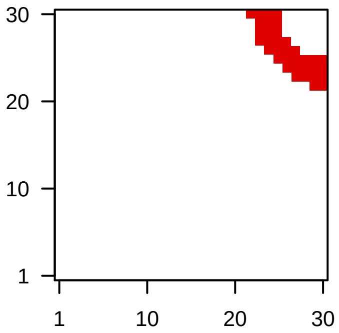

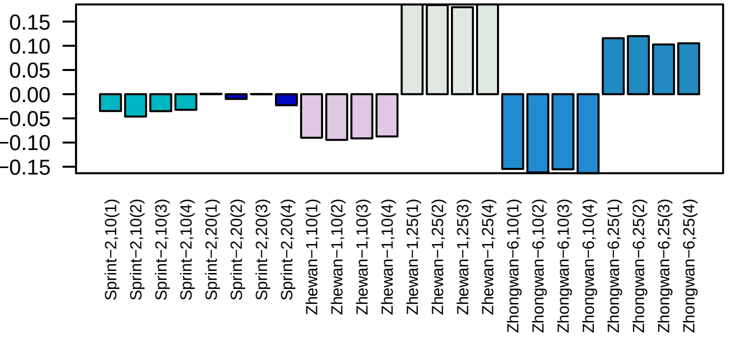

Spot Genelist

Rank	ID	max e	min e	r	Symbol	Description
1	TRINITY_DN8	0.95	−0.26	0.42		
2	comp40422_c	0.82	−0.36	0.64		
3	TRINITY_DN5	0.8	−0.15	0.64		
4	TRINITY_DN6	0.76	−0.52	0.36		
5	TRINITY_DN1	0.65	−0.13	0.44		
6	TRINITY_DN5	0.64	−0.35	0.65		
7	TRINITY_DN1	0.64	−0.33	0.51		
8	TRINITY_DN4	0.63	−0.28	0.61		
9	TRINITY_DN1	0.6	−0.37	0.68		
10	TRINITY_DN3	0.58	−0.11	0.67		
11	TRINITY_DN6	0.56	−0.31	0.66		
12	TRINITY_DN7	0.55	−0.17	0.7		
13	TRINITY_DN2	0.52	−0.15	0.74		
14	TRINITY_DN7	0.52	−0.24	0.58		
15	TRINITY_DN2	0.51	−0.18	0.35		
16	TRINITY_DN7	0.5	−0.37	0.79		
17	TRINITY_DN1	0.5	−0.58	0.67		
18	TRINITY_DN7	0.5	−0.28	0.43		
19	TRINITY_DN9	0.5	−0.41	0.38		
20	TRINITY_DN1	0.5	−0.21	0.89		

K−Means Cluster

Spot Summary: K

metagenes = 122
genes = 183

<r> metagenes = 0.86
<r> genes = 0.47
beta: r2= 0.1 / log p= −0.87

samples with spot = 0 (0 %)

Overview Map

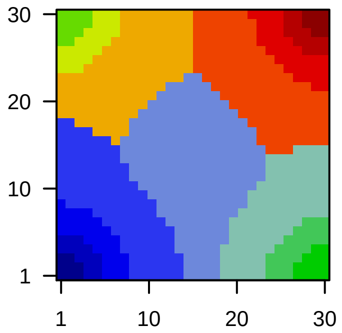

Spot

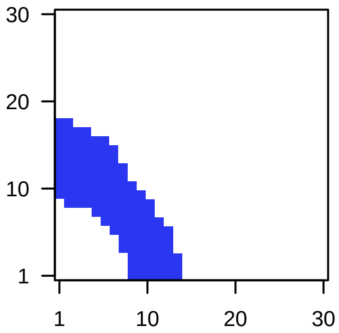

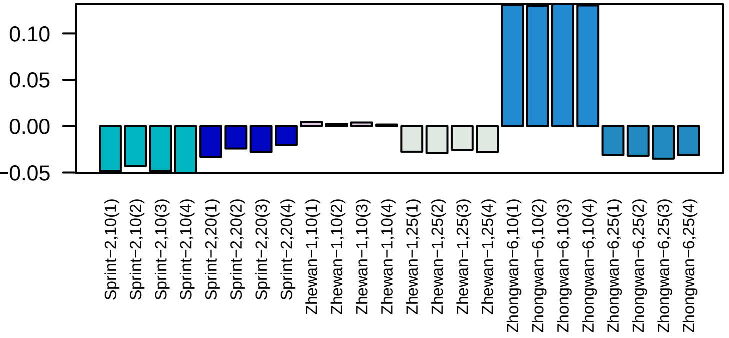

Spot Genelist

Rank	ID	max e	min e	r	Symbol	Description
1	TRINITY_DN9	1.02	−1.15	0.21		
2	TRINITY_DN1	0.57	−0.28	0.58		
3	TRINITY_DN4	0.55	−0.41	0.51		
4	TRINITY_DN3	0.48	−0.3	0.55		
5	TRINITY_DN1	0.45	−0.52	0.08		
6	TRINITY_DN9	0.43	−0.44	0.12		
7	TRINITY_DN1	0.41	−0.06	0.36		
8	TRINITY_DN1	0.4	−0.22	0.56		
9	TRINITY_DN1	0.4	−0.41	0.48		
10	TRINITY_DN1	0.39	−0.18	0.47		
11	TRINITY_DN2	0.37	−0.21	0.53		
12	TRINITY_DN3	0.35	−0.26	0.47		
13	TRINITY_DN3	0.35	−0.05	0.74		
14	TRINITY_DN5	0.33	−0.12	0.81		
15	TRINITY_DN1	0.33	−0.08	0.73		
16	TRINITY_DN2	0.32	−0.09	0.74		
17	TRINITY_DN1	0.32	−0.22	0.62		
18	TRINITY_DN2	0.32	−0.07	0.83		
19	TRINITY_DN1	0.3	−0.1	0.89		
20	TRINITY_DN8	0.3	−0.14	0.83		

K−Means Cluster

Spot Summary: L

metagenes = 217
genes = 671

<r> metagenes = 0.14
<r> genes = 0.07
beta: r2= 0 / log p= −0.1

samples with spot = 0 (0 %)

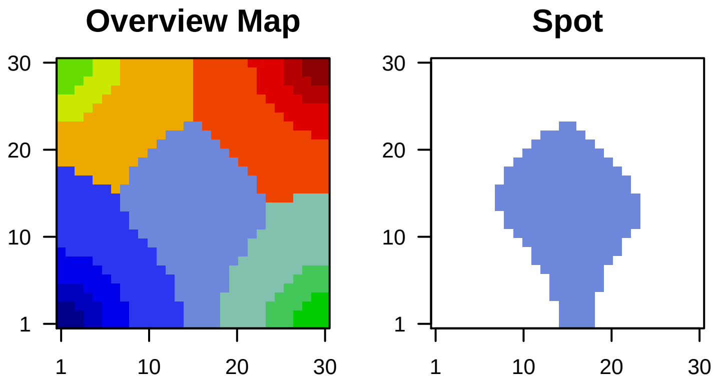

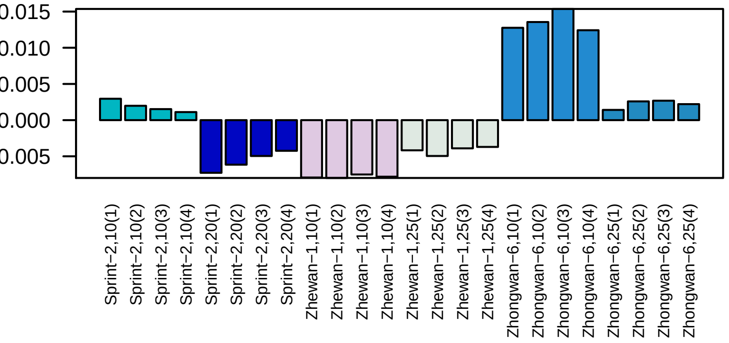

Spot Genelist

Rank	ID	max e	min e	r	Symbol	Description
1	TRINITY_DN7	0.92	−1.05	0.54		
2	TRINITY_DN1	0.47	−0.04	0.47		
3	TRINITY_DN5	0.45	−0.46	0.64		
4	TRINITY_DN5	0.44	−0.41	0.23		
5	TRINITY_DN5	0.3	−0.14	0.43		
6	TRINITY_DN1	0.25	−0.11	0.81		
7	TRINITY_DN3	0.25	−0.21	0.64		
8	TRINITY_DN1	0.24	−0.2	0.05		
9	TRINITY_DN9	0.23	−0.16	0.46		
10	TRINITY_DN3	0.23	−0.13	0.66		
11	TRINITY_DN1	0.21	−0.14	0.69		
12	TRINITY_DN4	0.21	−0.26	0.42		
13	TRINITY_DN1	0.21	−0.18	0.78		
14	TRINITY_DN5	0.21	−0.2	0.64		
15	TRINITY_DN2	0.21	−0.15	0.07		
16	TRINITY_DN5	0.2	−0.19	0.68		
17	TRINITY_DN1	0.2	−0.09	0.59		
18	TRINITY_DN5	0.19	−0.23	0.53		
19	comp6713_c0	0.19	−0.11	0.4		
20	comp39208_c	0.19	−0.03	0.05		

K−Means Cluster

Spot Summary: M

metagenes = 8
genes = 56

<r> metagenes = 1
<r> genes = 0.77
beta: r2= 8.04 / log p= −Inf

samples with spot = 8 (33.3 %)
Zhewan−1(10) : 4 (100 %)
Zhongwan−6(10) : 4 (100 %)

Overview Map

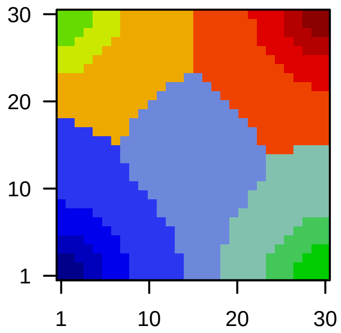

Spot

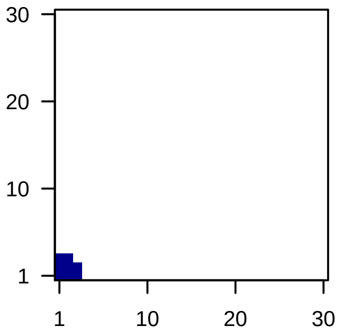

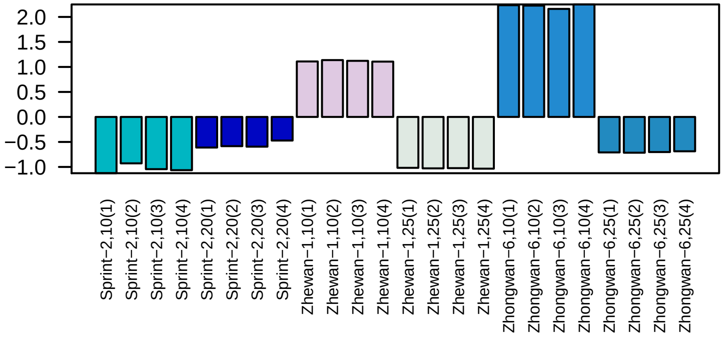

Spot Genelist

Rank	ID	max e	min e	r	Symbol	Description
1	TRINITY_DN1	13.12	−4.78	0.73		
2	TRINITY_DN7	11.07	−3.03	0.95		
3	TRINITY_DN1	8.39	−3.05	0.73		
4	TRINITY_DN1	6.17	−3.24	0.93		
5	TRINITY_DN4	5.77	−4.28	0.74		
6	TRINITY_DN3	5.76	−2.29	0.92		
7	TRINITY_DN1	5.25	−2.61	0.94		
8	TRINITY_DN1	4.57	−1.48	0.97		
9	TRINITY_DN2	4.53	−1.15	0.86		
10	TRINITY_DN3	4.41	−2.6	0.97		
11	TRINITY_DN2	4.05	−1.09	0.8		
12	TRINITY_DN3	3.87	−6.3	0.72		
13	TRINITY_DN3	3.49	−1.03	0.93		
14	TRINITY_DN7	3.48	−1.29	0.99		
15	TRINITY_DN2	3.3	−0.56	0.8		
16	TRINITY_DN2	3.29	−0.85	0.52		
17	TRINITY_DN1	3.26	−3.12	0.73		
18	TRINITY_DN1	2.87	−1.41	0.58		
19	TRINITY_DN2	2.8	−1.04	0.95		
20	TRINITY_DN8	2.78	−0.81	0.9		

K−Means Cluster

Spot Summary: N

metagenes = 14
genes = 33

<r> metagenes = 0.99
<r> genes = 0.72
beta: r2= 1.99 / log p= −Inf

samples with spot = 4 (16.7 %)
Zhongwan−6(10) : 4 (100 %)

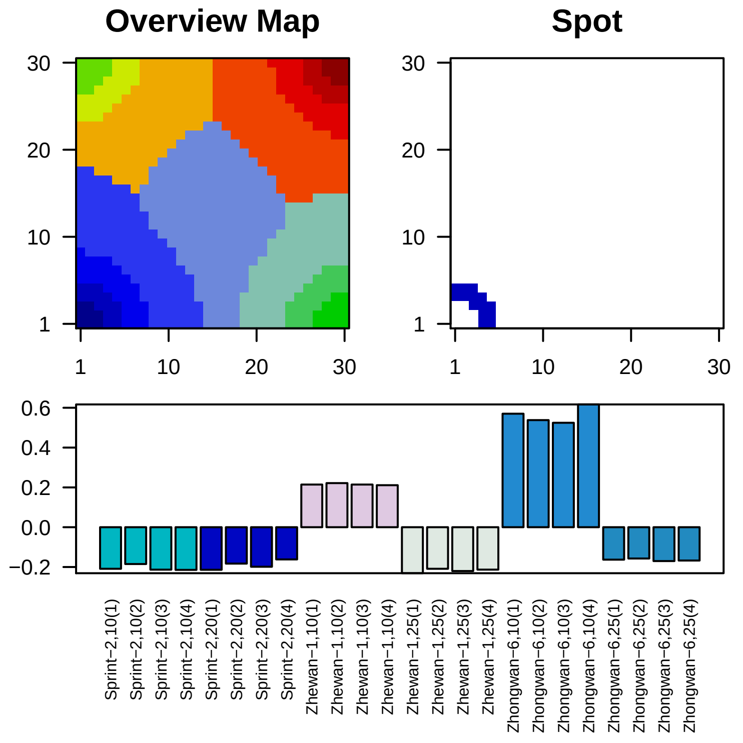

Spot Genelist

Rank	ID	max e	min e	r	Symbol	Description
1	comp6342_c0	1.55	−0.26	0.86		
2	TRINITY_DN2	1.37	−0.93	0.44		
3	TRINITY_DN1	1.31	−0.22	0.85		
4	TRINITY_DN1	1.11	−0.48	0.89		
5	TRINITY_DN9	1.03	−0.52	0.91		
6	TRINITY_DN1	0.99	−0.32	0.98		
7	TRINITY_DN6	0.95	−0.14	0.84		
8	TRINITY_DN1	0.94	−0.25	0.95		
9	TRINITY_DN3	0.94	−0.4	0.89		
10	TRINITY_DN4	0.9	−0.8	0.69		
11	TRINITY_DN3	0.89	−0.3	0.67		
12	TRINITY_DN2	0.88	−0.28	0.87		
13	TRINITY_DN9	0.86	−0.25	0.91		
14	TRINITY_DN2	0.85	−0.17	0.92		
15	TRINITY_DN4	0.84	−0.42	0.94		
16	TRINITY_DN5	0.84	−0.14	0.84		
17	TRINITY_DN1	0.81	−0.23	0.9		
18	TRINITY_DN1	0.79	−0.16	0.88		
19	TRINITY_DN1	0.78	−0.23	0.96		
20	TRINITY_DN1	0.73	−0.26	0.89		

K−Means Cluster

Spot Summary: O

metagenes = 32
genes = 111

<r> metagenes = 0.97
<r> genes = 0.61
beta: r2= 0.59 / log p= −4.96

samples with spot = 0 (0 %)

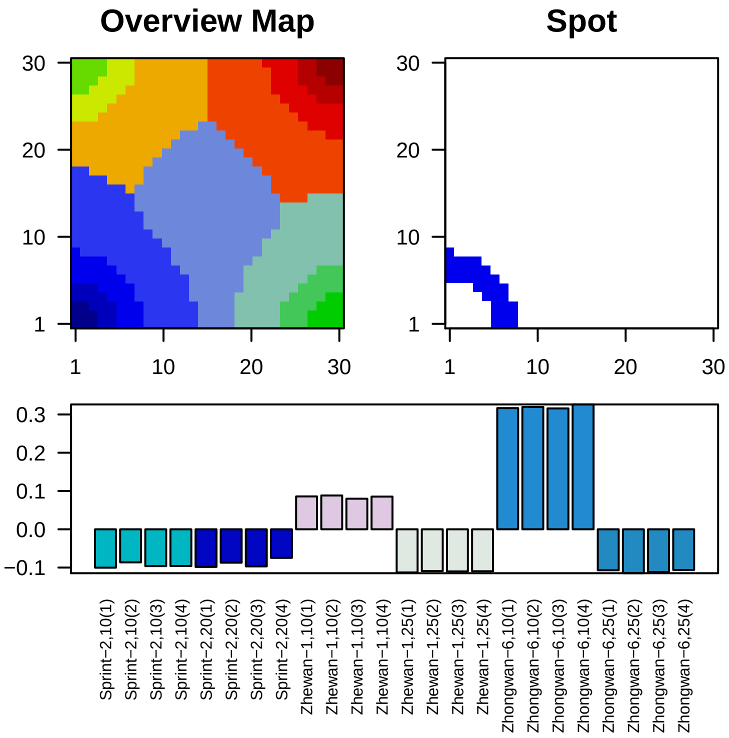

Spot Genelist

Rank	ID	max e	min e	r	Symbol	Description
1	TRINITY_DN1	0.96	−0.58	0.57		
2	TRINITY_DN5	0.89	−0.53	0.5		
3	comp47596_c	0.89	−0.13	0.86		
4	TRINITY_DN1	0.83	−0.27	0.73		
5	TRINITY_DN1	0.83	−0.46	0.82		
6	TRINITY_DN3	0.75	−0.48	0.54		
7	TRINITY_DN9	0.73	−0.46	0.53		
8	TRINITY_DN5	0.71	−0.31	0.51		
9	comp73378_c	0.69	−0.17	0.71		
10	TRINITY_DN6	0.68	−0.59	0.56		
11	TRINITY_DN2	0.68	−0.17	0.93		
12	TRINITY_DN8	0.68	−0.43	0.8		
13	TRINITY_DN1	0.65	−0.13	0.8		
14	TRINITY_DN4	0.65	−0.3	0.91		
15	TRINITY_DN1	0.65	−0.32	0.58		
16	TRINITY_DN4	0.65	−0.36	0.47		
17	TRINITY_DN2	0.64	−0.44	0.31		
18	TRINITY_DN3	0.64	−0.28	0.85		
19	TRINITY_DN2	0.64	−0.1	0.88		
20	TRINITY_DN2	0.61	−0.43	0.78		
